# Supplementary material for: Pterostilbene protects cochlea from ototoxicity in streptozotocin-induced diabetic rats by inhibiting apoptosis
Source: PLoS One. 2020 Jul 28;15(7):e0228429. doi: 10.1371/journal.pone.0228429 (PMC7386625; doi:10.1371/journal.pone.0228429)
Supplement: S1 File — (PDF) [file pone.0228429.s001.pdf]

Evrak Tarih ve Sayısı: 20/09/2019-E.76864

T.C.
MANİSA CELAL BAYAR ÜNİVERSİTESİ
Tıp Fakültesi Dekanlığı
Hayvan Deneyleri Yerel Etik Kurulu

Sayı : 77637435-050.04.04-
Konu : Etik Kurul Kararı-Seren Gülşen Gürgen - Deneysel diyabet

Sayın Doç. Dr. Seren Gülşen GÜRGEN

Dilekçeniz ile ilgili Etik Kurul kararı ektedir. Bilgilerinizi rica ederim.

e-imzalıdır
Prof. Dr. Ercüment ÖLMEZ
Kurul Başkanı

Ek: 17.09.2019 KARAR TUTANAĞI (1 sayfa)

Adres:Manisa Celal Bayar Üniversitesi Uncubozköy Kampüsü Manisa
Telefon:(0 236) 2338586 Faks(0 236) 2331466
E-Posta:tip@cbu.edu.tr Elektronik Ağ:http://tip.cbu.edu.tr
Bilgi İçin:Ruhsar Gürel
Unvanı: Sekreter

BELC5RH02
Bu belge 5070 sayılı Elektronik İmza Kanununun 5. Maddesi gereğince güvenli elektronik imza ile imzalanmıştır.

T.C.
Manisa Celal Bayar Üniversitesi
Hayvan Deneyleri Yerel Etik Kurulu

**KARAR TARİH / NO**	17/ 09 / 2019 / 77.637.435			
**ARAŞTIRMANIN ADI**	Deneysel Diyabet Hayvan Modelinde Pterostilben'nin Kokleaya Etkisinin Araştırılması			
**SORUMLU ARAŞTIRMACI**	Doç. Dr. S. Gülşen GÜRGEN			
**ARAŞTIRMA EKİBİ**	Dr. Öğr. Üyesi Sibel Özdaş,- Doç. Dr. Talih Özdaş,- Arş. Gör. Bora Taştekin			
**ARAŞTIRMANIN NİTELİĞİ**	**UZMANLIK TEZİ** ☐	**YÜKSEK LİSANS--DOKTORA TEZİ** ☐	**AKADEMİK AMAÇLI** ☒	Eğitim ☐
**DEĞERLENDİRİLEN BELGELER**	16 / 09 / 2019 / Tarih ve sayılı; dilekçe			
**KARAR BİLGİLERİ**	Dilekçe incelenmiş, bilimsel ve etik açıdan **UYGUN** olduğuna oy birliği ile karar verilmiştir.			

Unvanı/Adı/Soyadı		Araştırma İle İlişkisi Olan Üye	Toplantıya Katılmayan Üye	Unvanı /Adı /Soyadı		Araştırma İle İlişkisi Olan Üye	Toplantıya Katılmayan Üye
Prof. Dr. Ercüment ÖLMEZ		☐	☐	Prof. Dr. Gökhan TEMELTAŞ	----------	☐	☒
Prof. Dr. İsmet TOPÇU<br>Anestezi ve Reanimasyon AD	--------	☐	☒	Prof. Dr. Özge YILMAZ<br>Çocuk Hastalıkları AD.		☐	☐
Prof. Dr. Ertuğrul TATLISUMAK<br>Anatomi AD		☐	☐	Dr. Öğr. Üyesi Fulya OCAK –<br>Veteriner		☐	☐
Prof. Dr. Kıvanç GÜNHAN<br>DEHAM MD	---------	☐	☒	Saime ÖZKARA<br>Sivil Toplum Üyesi		☐	☐
Dr. Öğr. Üyesi Selim ALTAN<br>Tıp Tarihi ve Etik AD		☐	☐	Levent TEZCAN<br>Sivil Üye		☐	☐

Etik Kurulumuzun kararı yukarda belirtilmiştir. Araştırma Başvuru Formunun Taahhütname kısmında belirtilmiş olan hususların dikkate alınarak istenilen bilgilerin Etik Kurulumuza zamanında iletilmesi konusunda bilgilerinizi ve gereğini rica ederim.

Ercüment ÖLMEZ
Başkan
